# Direct Oral Anti-Xa Anticoagulants and the Future of Factor XI/FXIa Inhibition: A New Paradigm in Thrombosis Prevention

**DOI:** 10.3390/pharmacy14010019

**Published:** 2026-01-27

**Authors:** Francesca Futura Bernardi, Dario Bianco, Rosaria Lanzillo, Natalia Diana, Mario Scarpato, Antonio Lalli, Aniello Corallo, Consiglia Riccardi, Ugo Trama, Alessandro Perrella, Manuela Basaglia, Ada Maffettone, Pierpaolo Di Micco, Carmine Siniscalchi

**Affiliations:** 1 Coordination of the Regional Healt System, General Directorate for Healt Protection, SIFO (“Società Italiana di Farmacia Ospedaliera e Dei Servizi Farmaceutici Delle Aziende Sanitarie”) Campania Section, 80100 Naples, Italy; francescafutura.bernardi@regione.campania.it (F.F.B.); ugo.trama@regione.campania.it (U.T.); 2 Pozzuoli Hospital, SIFO (“Società Italiana di Farmacia Ospedaliera e Dei Servizi Farmaceutici Delle Aziende Sanitarie”) Campania Section, 80100 Naples, Italy; dario.bianco@aslnapoli2nord.it; 3 University Hospital “Federico II”, SIFO (“Società Italiana di Farmacia Ospedaliera e Dei Servizi Farmaceutici Delle Aziende Sanitarie”) Campania Section, 80100 Naples, Italy; rosaria.lanzillo@unina.it; 4 Local Healt Unit, SIFO (“Società Italiana di Farmacia Ospedaliera e Dei Servizi Farmaceutici Delle Aziende Sanitarie”) Campania Section, 80100 Naples, Italy; natadiana7@gmail.com; 5 Monaldi Hospital, SIFO (“Società Italiana di Farmacia Ospedaliera e Dei Servizi Farmaceutici Delle Aziende Sanitarie”) Campania Section, 80100 Naples, Italy; mario.scarpato@ospedalideicolli.it; 6 Fatebenefratelli Hospital, SIFO (“Società Italiana di Farmacia Ospedaliera e Dei Servizi Farmaceutici Delle Aziende Sanitarie”) Campania Section, 80100 Naples, Italy; lalli.antonio@fbfna.it; 7 Local Healt Authority of Salerno, SIFO (“Società Italiana di Farmacia Ospedaliera e Dei Servizi Farmaceutici Delle Aziende Sanitarie”) Campania Section, 84131 Salerno, Italy; nellocorallo1986@gmail.com; 8 Regional Center of Pharmacovigilance and Pharmacoepidemiology, SIFO (“Società Italiana di Farmacia Ospedaliera e Dei Servizi Farmaceutici Delle Aziende Sanitarie”) Campania Section, University of Campania Luigi Vanvitelly, 80100 Naples, Italy; consigliariccardi@icloud.com; 9 First Division of Infectious Disease, P.O. Cotugno, AO dei Colli, 80131 Naples, Italy; alessandro.perrella@ospedalideicolli.it; 10 Department of Internal Medicine, Parma University Hospital, 43125 Parma, Italy; mbasaglia80@gmail.com (M.B.); csiniscalchi84@gmail.com (C.S.); 11 Department of Internal Medicine, San Luca Vallo della Lucania, 84131 Salerno, Italy; adamaff@hotmail.com; 12 Interal Medicine Ward, “P.O. Santa Maria delle Grazie”, 80076 Naples, Italy

**Keywords:** direct oral anticoagulants, factor Xa inhibitors, factor XI inhibition, FXIa inhibitors, milvexian, asundexian, abelacimab, thrombosis prevention, bleeding risk, cancer-associated thrombosis

## Abstract

The introduction of direct oral anticoagulants (DOACs), particularly factor Xa (FXa) inhibitors, has transformed the prevention and treatment of thromboembolic events. These agents have largely replaced vitamin K antagonists across most indications due to their predictable pharmacokinetics, reduced rates of intracranial bleeding, and overall ease of use. Nevertheless, a substantial residual bleeding risk remains, particularly gastrointestinal bleeding and clinically relevant non-major bleeding in elderly, frail, or polymedicated patients. Furthermore, the management of patients with severe renal dysfunction, active cancer, especially gastrointestinal or genitourinary malignancies and those requiring complex pharmacological regimens, continues to pose significant challenges. These limitations have intensified interest in targeting earlier steps of the coagulation cascade, specifically factor XI (FXI) and its activated form (FXIa). FXI occupies a unique mechanistic position: it contributes substantially to pathological thrombosis while playing only a limited role in physiological hemostasis. Genetic, observational, and mechanistic evidence consistently demonstrates that FXI deficiency confers protection against venous thromboembolism and cardiovascular events while causing minimal spontaneous bleeding. This biological paradigm has catalyzed the development of novel FXI/FXIa inhibitors, including small-molecule agents (asundexian, milvexian) and biological therapies (abelacimab). Clinical trials such as AXIOMATIC-TKR, PACIFIC-AF, and OCEANIC-AF, and ongoing programmes including ASTER and MAGNOLIA suggest that FXI inhibition may preserve antithrombotic efficacy while substantially reducing bleeding risk. This review summarizes the current landscape of oral FXa inhibitors, outlines the biological rationale for FXI/FXIa inhibition, and discusses the evolving clinical evidence supporting what may represent the next major advance in anticoagulant therapy.

## 1. Introduction

**Figure 1:**
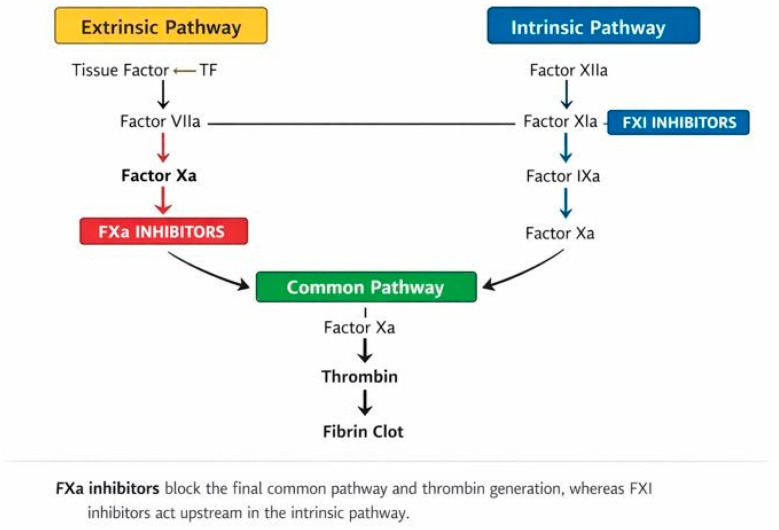
Mechanistic Position of FXa and FXI/FXIa Inhibitors within the Coagulation Cascade. Legend. Simplified representation of the coagulation cascade showing the extrinsic (tissue factor–dependent) and intrinsic (contact activation) pathways. FXa inhibitors block the final common pathway and suppress thrombin generation, whereas FXI/FXIa inhibitors act upstream in the intrinsic amplification loop, selectively attenuating pathological thrombosis while largely preserving physiological haemostasis.

Thromboembolic disorders remain a leading cause of morbidity and mortality worldwide, representing a substantial global health burden [1]. Atrial fibrillation (AF) predisposes patients to cardioembolic stroke, while venous thromboembolism (VTE), encompassing deep vein thrombosis and pulmonary embolism, continues to impose a major clinical and economic impact. Effective anticoagulation is therefore essential across multiple clinical contexts, yet achieving the optimal balance between preventing thrombosis and minimizing bleeding remains challenging. Vitamin K antagonists (VKAs) dominated anticoagulant therapy for decades despite several limitations, including high interpatient variability, a narrow therapeutic window, the need for frequent laboratory monitoring, dietary interactions, and substantial bleeding risk. The introduction of direct oral anticoagulants (DOACs) transformed clinical practice over the past decade. Among these, factor Xa (FXa) inhibitors such as apixaban, rivaroxaban, and edoxaban rapidly became first-line therapies for AF and VTE due to their predictable pharmacokinetics, reduced monitoring requirements, and favorable safety profiles [2,3]. However, DOACs do not fully embody the characteristics of an “ideal anticoagulant”. Important unmet needs persisted, particularly in populations with multiple comorbidities or those underrepresented in pivotal trials. Elderly patients with impaired or fluctuating glomerular filtration rate (GFR), individuals exposed to polypharmacy, oncologic patients, and frail subjects often present specific challenges to maintaining safe and effective DOAC therapy [4]. These limitations have fueled growing interest in a new anticoagulation paradigm targeting factor XI (FXI) and its activated form FXIa (Figure 1). Positioned strategically within the intrinsic coagulation pathway, FXI plays a pivotal role in thrombosis but is less essential for physiological hemostasis. Therefore, its inhibition offers the potential to dissociate antithrombotic efficacy from bleeding risk. This concept of “safer anticoagulation” has generated considerable scientific and clinical interest, supported by mechanistic studies and emerging evidence from early-phase clinical trials [5]. This review provides a focused discussion on the current role and limitations of FXa inhibitors and examines in brief the scientific rationale and emerging evidence that support FXI/FXIa inhibition as the next frontier in anticoagulation. The mechanistic position of FXa and FXI within the coagulation cascade is illustrated in Figure 1, highlighting the distinct biological rationale underlying FXI-targeted anticoagulation. In recent years, several phase II and phase III clinical programs have explored the concept of FXI/FXIa inhibition as a novel anticoagulant strategy, including AXIOMATIC-TKR, AXIOMATIC-SSP, PACIFIC-AF, OCEANIC-AF, and AZALEA-TIMI 71. These studies consistently showed marked reductions in bleeding compared with FXa inhibitors, with variable efficacy depending on the thrombotic setting. The purpose of this narrative review is to critically compare FXa inhibition with FXI/FXIa inhibition from mechanistic, clinical, and pharmacoepidemiologic perspectives, with a focus on high-risk patient populations and future therapeutic positioning.

## 2. What We Learned from DOACs Xa-Inhibitors?

In recent years, DOAC Xa-inhibitors (i.e., rivaroxaban, apixaban, edoxaban) have profoundly changed the clinical landscape of long-term anticoagulation, particularly for the primary prevention of cardioembolic stroke in atrial fibrillation (AF) and the secondary prevention of venous thromboembolism (VTE) [6,7,8]. Phase III clinical trials in these settings consistently demonstrated not only the non-inferiority of Xa-inhibitors compared with warfarin regarding prevention of thromboembolic events but also their superior safety profile, especially with respect to intracranial haemorrhage [9]. Beyond AF and VTE, expanded clinical development programmes identified additional therapeutic opportunities. Evidence supports their use in patients with acute coronary syndrome complicated by AF, in peripheral artery disease, across different stages of claudication and even in portal vein thrombosis associated with liver cirrhosis [10,11]. Real-world data have highlighted both strengths and limitations. While substantial reductions in intracranial hemorrhage have been confirmed outside of clinical trial settings, increases in occult bleeding and off-label prescribing (e.g., in valvular AF or cerebral venous thrombosis) have generated heterogeneous and sometimes conflicting clinical evidence [12]. These shortcomings represent critical starting points for the development of next-generation anticoagulants, namely, agents targeting factor XI/XIa. From a pathophysiological standpoint, FXa lies at the convergence of the intrinsic and extrinsic pathways and directly controls the conversion of prothrombin to thrombin. Inhibition of FXa therefore suppresses both pathological thrombus formation and physiological hemostasis, explaining the observed reduction in ischemic events together with a persistent risk of gastrointestinal and intracranial bleeding. In contrast, FXI primarily amplifies thrombin generation through the intrinsic pathway, which is especially activated in inflammatory, cancer-related, and contact-mediated thrombosis. This biological distinction underlies the hypothesis that FXI/FXIa inhibition may preserve antithrombotic efficacy while minimizing bleeding.

## 3. Clinical Settings with High Unmet Needs

A major driver of interest in FXI/FXIa inhibitors is the significant proportion of patients who require anticoagulation but either do not tolerate or inadequately respond to currently available agents. Several scenarios illustrate where FXI inhibition may provide meaningful advances.

### 3.1. Cancer-Associated Thrombosis

Patients with active cancer represent one of the most challenging groups to manage with anticoagulation. Recurrent VTE risk remains high due to tumor-driven hypercoagulability, immobility, inflammation, and cancer-directed therapies. Simultaneously, many malignancies, particularly gastrointestinal and genitourinary tumors, predispose individuals to bleeding, complicating the use of DOACs and VKAs. FXI inhibitors, which exert antithrombotic effects while exerting minimal interference with primary hemostasis, may reduce bleeding without compromising VTE protection. Long-acting biological agents may also be advantageous for patients undergoing chemotherapy who struggle with oral adherence [13,14]. In randomized trials of DOACs in cancer-associated thrombosis, major bleeding occurred in approximately 6–8% of patients at 6 months, with gastrointestinal bleeding rates exceeding 10% in patients with gastrointestinal malignancies. In the SELECT-D trial, rivaroxaban reduced recurrent VTE compared with dalteparin (4% vs. 11%) but increased major bleeding (6% vs. 4%) and clinically relevant non-major bleeding (13% vs. 4%). These figures highlight the narrow therapeutic margin of FXa inhibitors in oncologic populations.

### 3.2. Older Adults and Frail Patients

Ageing is associated with endothelial dysfunction, polypharmacy, declining renal function, increased fall risk, and greater vulnerability to both thrombosis and bleeding. In older adults, DOACs often require dose adjustments, and drug–drug interactions become clinically meaningful. FXI inhibition, which is less dependent on renal clearance and largely unaffected by common pharmacological interactions, may offer safer long-term anticoagulation options for this high-risk group [15]. In real-world registries, patients aged ≥80 years treated with FXa inhibitors experience major bleeding rates of 3–5% per year and clinically relevant non-major bleeding exceeding 10% per year, particularly in the presence of renal impairment and polypharmacy. Although DOACs reduce intracranial hemorrhage by approximately 50% compared with VKAs, overall bleeding remains a major cause of treatment discontinuation in frail populations.

### 3.3. Chronic Kidney Disease

Patients with advanced chronic kidney disease (CKD) frequently cannot receive VKAs or DOACs safely due to altered pharmacokinetics and markedly increased bleeding risk. Because monoclonal antibodies targeting FXI are cleared independently of renal function, they may provide more predictable and stable anticoagulation in this population [16]. If efficacy is confirmed in phase III trials, FXI inhibitors could address one of the most persistent unmet needs in anticoagulation care. In patients with advanced CKD (eGFR < 30 mL/min), DOAC exposure increases by 1.5–2-fold and bleeding rates can exceed 10% per year, limiting their safe use. Because monoclonal FXI inhibitors are not cleared by the kidney, their pharmacokinetics remain stable across all stages of renal dysfunction, providing a strong theoretical advantage in this population.

### 3.4. History of Major Bleeding

Patients with a prior intracranial or major gastrointestinal bleed are often denied standard anticoagulation, despite ongoing thrombotic risk. FXI inhibitors may represent a paradigm shift, offering substantially lower bleeding risk while maintaining antithrombotic activity. Although additional data are needed, early studies consistently demonstrate extremely low rates of major bleeding with FXI-directed therapies [17]. In summary, FXI inhibition appears particularly well suited for patients situated at the intersection of high thrombotic and high bleeding risk, an area where current anticoagulants frequently fall short.

### 3.5. Antiphospholipid Syndrome (APS)

Patients with high-risk antiphospholipid syndrome, particularly those with triple-positive antiphospholipid antibodies, represent another group in which vitamin K antagonists remain the standard of care and DOACs are discouraged due to excess arterial and venous thrombotic events [18,19]. In this setting, FXI/FXIa inhibition represents a mechanistically attractive but still exploratory option. Because APS-associated thrombosis is driven by inflammation, complement activation, and contact pathway amplification, FXI inhibition could theoretically attenuate thrombus propagation without increasing bleeding. However, at present, no clinical data are available in APS, and its role remains speculative pending dedicated trials.

## 4. Biological and Clinical Rationale for Targeting FXI/FXIa

The search for safer anticoagulation has increasingly turned toward factor XI (FXI) and its activated form FXIa because of their distinctive roles within the coagulation cascade (Figure 1). As shown in Figure 1, FXI lies upstream within the intrinsic amplification loop of coagulation, in contrast to FXa, which directly controls thrombin generation. FXI functions primarily within the intrinsic, or contact activation, pathway. Unlike factor Xa and thrombin, as central enzymes required for both normal hemostasis and pathological thrombus formation, FXI mainly contributes to the amplification phase of coagulation rather than the initiation of clotting [14]. This distinction has important clinical implications: while FXa inhibition effectively prevents thromboembolic events, it simultaneously impairs physiological hemostasis and increases bleeding risk. In contrast, attenuating FXI activity may limit thrombosis without substantially compromising normal clot formation.

FXI is activated via a dual mechanism involving factor XIIa and a positive feedback loop mediated by thrombin. Once converted to FXIa, it enhances the generation of factor IXa, amplifying thrombin production. This promotes fibrin formation and stabilises thrombi, especially in settings characterized by sustained vascular injury or inflammation. Importantly, the intrinsic pathway is not essential for routine primary hemostasis, as demonstrated by individuals with hereditary FXI deficiency, who typically only exhibit mild or procedure-specific bleeding tendencies, most commonly in tissues with high fibrinolytic activity such as the oral cavity or urinary tract.

Epidemiological studies have shown that individuals with naturally low FXI levels have markedly reduced risks of venous thromboembolism and cardiovascular events [15]. These findings support the notion that FXI plays a larger role in pathological thrombosis relative to its limited involvement in everyday hemostasis. Mechanistic data further suggest that FXI may be particularly relevant in thrombosis associated with inflammatory states, malignancy, and atherosclerotic disease, conditions in which coagulation activation is heightened despite an intact tissue factor pathway [20].

Collectively, these observations provide the scientific foundation for the concept of “anticoagulation with less bleeding.” Targeting FXI/FXIa may:

- Suppress thrombin amplification while preserving baseline hemostatic mechanisms;
- Reduce major and clinically relevant bleeding;
- Minimize dependance on renal clearance;
- Offer a more favorable risk–benefit profile for high-risk groups such as cancer patients, older adults, and individuals with chronic kidney disease.

For these reasons, FXI and FXIa have emerged as highly promising targets for the next generation of anticoagulant therapies. Across phase II studies, FXI/FXIa inhibitors have consistently demonstrated a 60–90% reduction in major and clinically relevant non-major bleeding compared with FXa inhibitors. In PACIFIC-AF, asundexian reduced bleeding by approximately 70% versus apixaban. In AZALEA–TIMI 71, abelacimab reduced major or clinically relevant non-major bleeding from 9.5% with rivaroxaban to 2.6%. In AXIOMATIC-TKR, milvexian achieved similar VTE prevention to enoxaparin, with major bleeding rates below 1%.

## 5. Clinical Evidence for FXI/FXIa Inhibition

The pharmacological and clinical characteristics of currently available FXa inhibitors and emerging FXI/FXIa inhibitors are summarized in Table 1 and Table 2. The development of FXI-directed anticoagulants includes two main therapeutic classes: small-molecule FXIa inhibitors and biological agents targeting FXI or FXIa. Together, these compounds have generated a growing clinical evidence base demonstrating the feasibility of FXI inhibition as an antithrombotic strategy that may offer superior safety compared with conventional anticoagulants.

### 5.1. Small-Molecule FXIa Inhibitors

#### 5.1.1. Milvexian

Milvexian is an oral, selective FXIa inhibitor that was evaluated across a broad clinical program that included orthopedic surgery, secondary stroke prevention, acute coronary syndromes, and atrial fibrillation. Early studies in patients undergoing total knee arthroplasty, primarily the AXIOMATIC-TKR trials, demonstrated that multiple milvexian doses effectively reduced postoperative venous thromboembolism with a very low incidence of major bleeding [21]. These findings support the concept that FXIa inhibition may preserve antithrombotic efficacy while maintaining a favorable bleeding profile.

In secondary stroke prevention, the AXIOMATIC-SSP trial reported mixed results. Milvexian produced potent suppression of FXIa activity and maintained an excellent safety profile, but its impact on reducing recurrent stroke was modest [22]. These observations suggest that in cerebrovascular diseases driven by large-artery atherosclerosis or cardioembolism, FXIa inhibition alone may be insufficient to fully match current standard-of-care therapies. Nonetheless, the consistently low bleeding rates across studies highlight a potential advantage of this mechanistic class.

Ongoing phase III trials in atrial fibrillation (LIBREXIA-AF) and acute coronary syndromes (LIBREXIA-ACS) will clarify the optimal clinical role of milvexian and whether FXIa inhibition can provide stroke and systemic embolism protection comparable to current FXa inhibitors [23].

#### 5.1.2. Asundexian

Asundexian is another oral FXIa inhibitor with rapid onset and predictable pharmacokinetics. Phase II trials, including PACIFIC-AF, reported substantially reduced bleeding events compared with apixaban, supporting its potential utility in patients for whom bleeding is a major therapeutic concern [17]. However, the phase III OCEANIC-AF trial was terminated early due to inferior efficacy in preventing stroke and systemic embolism relative to standard anticoagulation.

These divergent outcomes from early and late phase trials underscore the complexity of cardioembolic stroke pathophysiology, which may rely more heavily on coagulation pathways not fully suppressed by FXIa inhibition. As summarized in Table 2, asundexian continues to be evaluated in other thrombotic conditions in which FXI-mediated amplification may play a more dominant role.

### 5.2. FXI Inhibitors

#### Abelacimab

Abelacimab is a fully human monoclonal antibody that binds both FXI and FXIa, producing sustained anticoagulation following a single monthly administration. Phase II evidence, particularly from the AZALEA-TIMI 71 trial, demonstrated profound and durable suppression of FXI activity with markedly lower bleeding rates compared with rivaroxaban [24]. This long-acting profile may be especially advantageous for patients with adherence difficulties or those requiring prolonged therapy.

Current clinical evaluation is centered on cancer-associated thrombosis, a setting with substantial unmet need, given the high recurrence risk and elevated bleeding potential in patients with gastrointestinal or genitourinary malignancies. Preliminary results suggest that abelacimab’s targeted mechanism and favorable bleeding profile may offer therapeutic benefits where DOACs or VKAs are poorly tolerated or contraindicated. Ongoing phase III studies will determine whether FXI inhibition can redefine anticoagulation standards in this population.

Together, small-molecule and biologic FXI inhibitors have established a strong scientific and clinical foundation: consistent evidence of reduced bleeding, preserved antithrombotic effects in many settings, and promising applicability in populations underserved by current anticoagulants.

Table 1 highlights the persistent limitations of FXa inhibitors, including renal clearance, bleeding risk, and drug–drug interactions. In contrast, Table 2 shows that FXI/FXIa inhibitors combine potent antithrombotic activity with reduced bleeding, renal-independent clearance, and simplified dosing, supporting their potential to address major unmet needs in anticoagulation.

## 6. Future Perspectives

The emergence of FXI/FXIa inhibitors raises the possibility of a new era in anticoagulation, one in which thrombotic risk can be significantly reduced without the substantial bleeding burden associated with current therapies. A structured comparison of the mechanistic, clinical, and practical differences between direct oral factor Xa inhibitors and factor XI/XIa inhibitors is provided in Table 3. Several themes shape the future direction of this developing field. Currently, FXa inhibitors account for more than 80% of the global oral anticoagulant market, reflecting their widespread adoption in atrial fibrillation and venous thromboembolism. Even a partial replacement by FXI/FXIa inhibitors in high-bleeding-risk populations would therefore represent a major shift in anticoagulant prescribing patterns.

If ongoing trials confirm that FXI inhibition consistently reduces bleeding while preserving antithrombotic efficacy, these agents may represent the closest approach to bleeding-sparing anticoagulation [25,26]. Such a shift could expand eligibility for long-term anticoagulation to patients currently considered too high-risk for standard therapy.

The availability of both oral and injectable FXI inhibitors may facilitate precision-based therapy tailored to individual patient characteristics. Long-acting injectable agents, for example, may be optimal for patients with poor adherence, whereas oral agents may remain preferable for those requiring rapid onset, easy titration, or straightforward reversibility. Moreover, the distinct pharmacologic properties of small-molecule versus biologic inhibitors could allow clinicians to match therapy to specific clinical vulnerabilities, such as severe renal impairment, active cancer, or prior bleeding history. Important questions remain before these agents can be fully integrated into routine practice. These include determining which clinical conditions rely most heavily on FXI-dependent thrombogenesis, establishing the long-term safety and immunogenicity of sustained FXI suppression, and defining peri-procedural management strategies as well as combinations with antiplatelet therapy. Economic considerations, particularly manufacturing costs and accessibility for patients with chronic disease, will also influence adoption and implementation. Addressing these challenges will be essential to realizing the full therapeutic potential of FXI inhibition.

## 7. Conclusions

Direct oral FXa inhibitors remain the cornerstone of contemporary anticoagulation, providing robust protection against thrombotic events with a more favorable safety profile than vitamin K antagonists. However, their limitations, particularly in elderly individuals, patients with cancer, those with renal impairment, and patients at high risk of bleeding, underscore important unmet clinical needs.

FXI/FXIa inhibition represents the most promising next step in anticoagulant therapy. Its unique mechanistic profile, rooted in the biology of the coagulation cascade, may enable effective thrombosis prevention while preserving essential hemostatic function. Early clinical trials of small-molecule inhibitors (such as asundexian and milvexian) and biological agents (such as abelacimab) show encouraging signals of reduced bleeding and acceptable efficacy, especially in populations traditionally considered difficult to treat.

Nevertheless, several limitations of FXa inhibitors will likely remain unresolved even with the introduction of FXIa-targeted agents. Patients with multiple comorbidities, complex pharmacological regimens, severe renal failure (including those with a glomerular filtration rate below 20 mL/min), or valvular atrial fibrillation may continue to present challenges. These persistent gaps highlight the ongoing need for research aimed at defining optimal anticoagulation strategies across heterogeneous and high-risk patient groups.

## Figures and Tables

**Table 1:** Comparative Characteristics of the Main Direct Oral FXa Inhibitors Used in Anticoagulation Therapy.

Feature	Apixaban	Rivaroxaban	Edoxaban
Pharmacokinetics	Predictable, low variability	Predictable, once-daily dosing	Predictable, once-daily dosing
Renal Clearance (%)	~27%	~35%	~50%
Hepatic clearance	Hepatic clearance	Hepatic clearance	Hepatic clearance
Dose adjustment (renal)	Dose adjustment (renal)	Dose adjustment (renal)	Dose adjustment (renal)
Dose adjustment (hepatic)	Dose adjustment (hepatic)	Dose adjustment (hepatic)	Dose adjustment (hepatic)
Major Advantages	Lower bleeding risk; Widely validated in elderly	Broad clinical indications; Strong VTE evidence	Lower major bleeding in selected populations
Major Limitations	Dose adjustments in frail patients	GI bleeding risk	Reduced efficacy in high body weight
Key Clinical Uses	AF stroke prevention; VTE treatment & secondary prevention	AF; VTE; PAD; selected ACS	AF; VTE treatment
Evidence Base	ARISTOTLE trial	ROCKET-AF, EINSTEIN, COMPASS	ENGAGE-AF, Hokusai-VTE

Legend. AF = atrial fibrillation; VTE = venous thromboembolism; PAD = peripheral artery disease; ACS = acute coronary syndrome. Dose adjustment is required for all FXa inhibitors in moderate to severe renal impairment and in hepatic dysfunction.

**Table 2:** Clinical and Pharmacologic Characteristics of Emerging FXI/FXIa Inhibitors.

Agent	Class	Mechanism of Action	Route/Dosing	Renal Clearance	Hepatic Clearance	Laboratory Monitoring	Key Evidence	Expected Advantages
Milvexian	Small-molecule FXIa inhibitor	Selective, reversible FXIa inhibition	Oral; once- or twice-daily	Minimal	CYP3A4 metabolism	Not routinely required	AXIOMATIC-TKR (VTE prevention), AXIOMATIC-SSP (stroke prevention)	Very low major bleeding; oral administration
Asundexian	Small-molecule FXIa inhibitor	Selective FXIa blockade	Oral	Minimal	CYP-mediated metabolism	Not required	PACIFIC-AF (reduced bleeding vs. apixaban)	Lower bleeding risk, simple pharmacokinetics
Abelacimab	Monoclonal antibody	Dual inhibition of FXI and FXIa	Monthly subcutaneous injection	None	None (reticulo-endothelial)	Not required	AZALEA-TIMI 71 (lower bleeding vs. rivaroxaban)	Long-acting action, renal-independent clearance

This table summarises the pharmacologic mechanisms, dosing strategies, and main clinical trial data for the three leading FXI/FXIa inhibitors under development (milvexian, asundexian, and abelacimab), highlighting their potential to reduce bleeding while maintaining antithrombotic efficacy. Unlike DOACs, monoclonal antibodies such as abelacimab are not cleared by the kidney or liver and do not require routine laboratory monitoring. Small-molecule FXIa inhibitors have partial hepatic metabolism but minimal renal dependence compared with FXa inhibitors.

**Table 3:** Comparison of DOACs (direct oral anti-Xa anticoagulants) versus FXI/FXIa inhibitors: advantages and disadvantages.

Domain	DOACs (Anti-Xa: Apixaban, Rivaroxaban, Edoxaban)	FXI/FXIa Inhibitors (Small Molecules: Asundexian, Milvexian; Biologics: Abelacimab, etc.)
Mechanistic target	Blocks FXa in the common pathway → strong anticoagulation but impacts physiological haemostasis	Blocks FXI/FXIa (intrinsic amplification loop) → aims to “decouple” thrombosis prevention from bleeding
Efficacy evidence base	Robust phase III evidence across AF and VTE; extensive real-world data	Evolving evidence (phase II/III); efficacy may be setting-dependent; some AF programmes showed limitations
Bleeding profile	Lower ICH vs. VKA, but residual bleeding persists (notably GI and CRNM bleeding)	Consistently lower major/CRNM bleeding signals in several trials vs. anti-Xa comparators (key rationale)
High-bleeding-risk patients	Still challenging in frail elderly, prior major bleeding, GI lesions/cancer	Potentially best fit population, pending definitive outcomes in each indication
Renal function	Relevant renal clearance (dose adjustments; concerns in advanced CKD)	Often minimal/none renal clearance (esp. mAbs) → theoretical advantage in advanced CKD
Drug–drug interactions	Clinically relevant interactions (P-gp/CYP3A4; polypharmacy issues)	Variable: small molecules may still involve CYP pathways; biologics usually few interactions
Dosing & adherence	Oral; once/twice daily depending on drug; adherence still critical	Oral (small molecules) similar adherence needs; monthly/long-acting injectables may improve adherence
Onset/offset & peri-procedural management	Well-defined peri-procedural strategies; relatively predictable offset	Peri-procedural protocols still being defined, especially for long-acting biologics
Monitoring	No routine monitoring; occasionally levels/anti-Xa in special scenarios	No routine monitoring expected; however, standardisation of assays/clinical pathways still in development
Reversal strategies	Established reversal options for anti-Xa (though access/cost may vary)	Specific reversal pathways may be limited/under development; long-acting agents raise practical questions
Clinical indications today	Broad, guideline-embedded indications (AF, VTE, other selected settings)	Indications not yet fully established; positioning likely in niches with high bleeding risk/unmet needs
Cancer-associated thrombosis	Effective but bleeding concerns in GI/GU cancers; careful selection needed	Attractive hypothesis (less bleeding; long-acting options) but confirmatory phase III needed
Long-term safety	Extensive long-term experience	Long-term safety (rare events, immunogenicity for biologics) still accruing
Cost & access	Generally established pricing and reimbursement pathways	May be higher cost initially; reimbursement/access uncertain until approved and positioned
Implementation in routine care	Familiar to clinicians; mature pathways (initiation, follow-up, switching)	Requires new pathways, education, and possibly new peri-procedural/bridging paradigms

This table summarises the main mechanistic, clinical, and practical differences between currently available direct oral factor Xa inhibitors (apixaban, rivaroxaban, edoxaban) and emerging factor XI/XIa inhibitors (including small molecules such as asundexian and milvexian, and monoclonal antibodies such as abelacimab). The comparison highlights their respective advantages and limitations across key domains, including haemostatic impact, bleeding risk, renal clearance, drug–drug interactions, dosing strategies, reversibility, and implementation in clinical practice. The table is intended to illustrate the potential role of FXI/XIa inhibitors as safer anticoagulant options in selected high-bleeding-risk populations while acknowledging the current robustness and breadth of evidence supporting DOAC therapy.

## Data Availability

All data generated or analysed in this study are included in this published article. Additional details may be provided by the corresponding author upon reasonable request.

## References

[1] Wendelboe A.M., Raskob G.E. (2016). Global Burden of Thrombosis: Epidemiologic Aspects. Circ. Res..

[2] Ruff C.T., Giugliano R.P., Braunwald E., Hoffman E.B., Deenadayalu N., Ezekowitz M.D., Camm A.J., Weitz J.I., Lewis B.S., Parkhomenko A. (2014). Comparison of the efficacy and safety of new oral anticoagulants with warfarin in patients with atrial fibrillation: A meta-analysis of randomised trials. Lancet.

[3] Prins M.H., Lensing A.W., Bauersachs R., van Bellen B., Bounameaux H., Brighton T.A., Cohen A.T., Davidson B.L., Decousus H., Raskob G.E. (2013). Oral rivaroxaban versus standard therapy for the treatment of symptomatic venous thromboembolism: A pooled analysis of the EINSTEIN-DVT and PE randomized studies. Thromb. J..

[4] Halperin J.L., Hankey G.J., Wojdyla D.M., Piccini J.P., Lokhnygina Y., Patel M.R., Breithardt G., Singer D.E., Becker R.C., Hacke W. (2014). Efficacy and safety of rivaroxdaban compared with warfarin among elderly patients with nonvalvular atrial fibrillation in the Rivaroxaban Once Daily, Oral, Direct Factor Xa Inhibition Compared With Vitamin K Antagonism for Prevention of Stroke and Embolism Trial in Atrial Fibrillation (ROCKET AF). Circulation.

[5] Paszek E., Undas A. (2025). Coagulation factor XI and coronary artery disease: Is there room for factor XI inhibitors?. Pol. Arch. Intern. Med..

[6] Granger C.B., Alexander J.H., McMurray J.J.V., Lopes R.D., Hylek E.M., Hanna M., Al-Khalidi H.R., Ansell J., Atar D., Avezum A. (2011). Apixaban versus warfarin in patients with atrial fibrillation. N. Engl. J. Med..

[7] Patel M.R., Mahaffey K.W., Garg J., Pan G., Singer D.E., Hacke W., Breithardt G., Halperin J.L., Hankey G.J., Piccini J.P. (2011). Rivaroxaban versus warfarin in nonvalvular atrial fibrillation. N. Engl. J. Med..

[8] Büller H.R., Décousus H., Grosso M.A., Mercuri M., Middeldorp S., Prins M.H., Raskob G.E., Schellong S.M., Schwocho L., Hokusai-VTE Investigators (2013). Edoxaban versus warfarin for the treatment of symptomatic venous thromboembolism. N. Engl. J. Med..

[9] Ruff C.T., Giugliano R.P., Braunwald E.A., Morrow D., Murphy S.A., Kuder J.F., Deenadayalu N., Jarolim P., Betcher J., Shi M. (2015). Association between edoxaban dose, concentration, anti-Factor Xa activity, and outcomes: An analysis of data from the randomised, double-blind ENGAGE AF-TIMI 48 trial. Lancet.

[10] Eikelboom J.W., Connolly S.J., Bosch J., Dagenais G.R., Hart R.G., Shestakovska O., Diaz R., Alings M., Lonn E.M., Anand S.S. (2017). Rivaroxaban with or without Aspirin in Stable Cardiovascular Disease. N. Engl. J. Med..

[11] Nagaoki Y., Aikata H., Daijyo K., Teraoka Y., Shinohara F., Nakamura Y., Hatooka M., Morio K., Nakahara T., Kawaoka T. (2018). Efficacy and safety of edoxaban for treatment of portal vein thrombosis following danaparoid sodium in patients with liver cirrhosis. Hepatol. Res..

[12] Steinberg B.A., Shrader P., Thomas L., Ansell J., Fonarow G.C., Gersh B.J., Kowey P.R., Mahaffey K.W., Naccarelli G., Reiffel J. (2016). Off-Label Dosing of Non-Vitamin K Antagonist Oral Anticoagulants and Adverse Outcomes: The ORBIT-AF II Registry. J. Am. Coll. Cardiol..

[13] Young A.M., Marshall A., Thirlwall J., Chapman O., Lokare A., Hill C., Hale D., Dunn J.A., Lyman G.H., Hutchinson C. (2018). Comparison of an Oral Factor Xa Inhibitor With Low Molecular Weight Heparin in Patients With Cancer With Venous Thromboembolism: Results of a Randomized Trial (SELECT-D). J. Clin. Oncol..

[14] Gailani D., Renné T. (2007). The intrinsic pathway of coagulation: A target for treating thromboembolic disease?. J. Thromb. Haemost..

[15] Preis M., Hirsch J., Kotler A., Zoabi A., Stein N., Rennert G., Saliba W. (2017). Factor XI deficiency is associated with lower risk for cardiovascular and venous thromboembolism events. Blood.

[16] Al-Horani R.A. (2020). Targeting factor XI(a) for anticoagulation therapy: A patent landscape. Pharm. Pat. Anal..

[17] Piccini J.P., Caso V., Connolly S.J., Fox K.A.A., Oldgren J., Jones W.S., Gorog D.A., Durdil V., Viethen T., Neumann C. (2022). Safety of the oral factor XIa inhibitor asundexian compared with apixaban in patients with atrial fibrillation (PACIFIC-AF): A multicentre, randomised, double-blind, double-dummy, dose-finding phase 2 study. Lancet.

[18] Pengo V., Denas G., Zoppellaro G., Jose S.P., Hoxha A., Ruffatti A., Andreoli L., Tincani A., Cenci C., Prisco D. (2018). Rivaroxaban vs warfarin in high-risk patients with antiphospholipid syndrome. Blood.

[19] Meroni P.L., Borghi M.O., Raschi E., Tedesco F. (2011). Pathogenesis of antiphospholipid syndrome: Understanding the antibodies. Nat. Rev. Rheumatol..

[20] Jordan K.R., Wyatt C.R., Fallon M.E., Woltjer R., Neuwelt E.A., Cheng Q., Gailani D., Lorentz C., Tucker E.I., McCarty O.J. (2022). Pharmacological reduction of coagulation factor XI reduces macrophage accumulation and accelerates deep vein thrombosis resolution in a mouse model of venous thrombosis. J. Thromb. Haemost..

[21] Weitz J.I., Strony J., Ageno W., Gailani D., Hylek E.M., Lassen M.R., Mahaffey K.W., Notani R.S., Roberts R., Segers A. (2021). Milvexian for the Prevention of Venous Thromboembolism. N. Engl. J. Med..

[22] Sharma M., Molina C.A., Toyoda K., Bereczki D., Bangdiwala S.I., Kasner S.E., Lutsep H.L., Tsivgoulis G., Ntaios G., Czlonkowska A. (2024). Safety and efficacy of factor XIa inhibition with milvexian for secondary stroke prevention (AXIOMATIC-SSP): A phase 2, international, randomised, double-blind, placebo-controlled, dose-finding trial. Lancet Neurol..

[23] Zhou W., Bozenhardt E., Alexander G.E., Zierhut M.L., Merali S., Ajavon-Hartmann A., Zannikos P., Back H., Suh E., Goyal N. (2025). Quantitative Model-Informed Dose Selection for a Milvexian Phase III Study in Patients with Atrial Fibrillation. Clin. Pharmacol. Ther..

[24] Patel S.M., Giugliano R.P., Morrow D.A., Goodrich E.L., Murphy S.A., Hug B., Parkar S., Chen S.-A., Goodman S.G., Joung B. (2025). Safety of Factor XI Inhibition with Abelacimab in Atrial Fibrillation by Kidney Function: A Prespecified Analysis of the AZALEA-TIMI 71 Randomized Clinical Trial. JAMA Cardiol..

[25] Ruff C.T., Patel S.M., Giugliano R.P., Morrow D.A., Hug B., Kuder J.F., Goodrich E.L., Chen S.-A., Goodman S.G., Joung B. (2025). Abelacimab versus Rivaroxaban in Patients with Atrial Fibrillation. N. Engl. J. Med..

[26] Mackman N., Bergmeier W., Stouffer G.A., Weitz J.I. (2020). Therapeutic strategies for thrombosis: New targets and approaches. Nat. Rev. Drug Discov..

